# Occupancy of red‐naped sapsuckers in a coniferous forest: using LiDAR to understand effects of vegetation structure and disturbance

**DOI:** 10.1002/ece3.1768

**Published:** 2015-11-02

**Authors:** Joseph D. Holbrook, Kerri T. Vierling, Lee A. Vierling, Andrew T. Hudak, Patrick Adam

**Affiliations:** ^1^ Department of Fish and Wildlife Sciences University of Idaho 875 Perimeter Drive MS 1136 Moscow Idaho 83844‐1136; ^2^ Department of Forest, Rangeland, and Fire Sciences University of Idaho 875 Perimeter Drive MS 1133 Moscow Idaho 83844‐1133; ^3^ Rocky Mountain Research Station Forest Service U.S. Department of Agriculture 1221 South Main Street Moscow Idaho 83843; ^4^ School of Mechanical and Materials Engineering Washington State University PO Box 642920 Pullman Washington 99164‐2920

**Keywords:** Conifer forest, discrete‐return LiDAR, Idaho, information theoretic, multimodel inference, occupancy models, *Sphyrapicus nuchalis*, woodpecker

## Abstract

Red‐naped sapsuckers (*Sphyrapicus nuchalis*) are functionally important because they create sapwells and cavities that other species use for food and nesting. Red‐naped sapsucker ecology within aspen (*Populus tremuloides*) has been well studied, but relatively little is known about red‐naped sapsuckers in conifer forests. We used light detection and ranging (LiDAR) data to examine occupancy patterns of red‐naped sapsuckers in a conifer‐dominated system. We surveyed for sapsuckers at 162 sites in northern Idaho, USA, during 2009 and 2010. We used occupancy models and an information‐theoretic approach to model sapsucker occupancy as a function of four LiDAR‐based metrics that characterized vegetation structure and tree harvest, and one non‐LiDAR metric that characterized distance to major roads. We evaluated model support across a range of territory sizes using Akaike's information criterion. Top model support was highest at the 4‐ha extent, which suggested that 4 ha was the most relevant scale describing sapsucker occupancy. Sapsuckers were positively associated with variation of canopy height and harvested area, and negatively associated with shrub and large tree density. These results suggest that harvest regimes and structural diversity of vegetation at moderate extents (e.g., 4 ha) largely influence occurrence of red‐naped sapsuckers in conifer forests. Given the current and projected declines of aspen populations, it will be increasingly important to assess habitat relationships, as well as demographic characteristics, of aspen‐associated species such as red‐naped sapsuckers within conifer‐dominated systems to meet future management and conservation goals.

## Introduction

Understanding factors that influence the distribution and occupancy of functionally important species is critical for ecosystem conservation and management. Red‐naped sapsuckers (*Sphyrapicus nuchalis*) are considered ecosystem engineers, and perhaps keystone species (Daily et al. 1993; Robles and Martin 2013). Ecosystem engineers are animals that directly or indirectly influence resources available to other species (Jones et al. 1994, 1997, 2010). Through their physical alteration of habitat, ecosystem engineers can form keystone structures (Tews et al. 2004; Remm and Lõhmus 2011), which are ecological features that increase biological diversity (e.g., beaver dams, animal burrows, woodpecker cavities). The activities of red‐naped sapsuckers influence the broader biodiversity of forest communities through creation of sapwells and excavation of cavities (Daily et al. 1993; Tews et al. 2004). Sapwells provide a food source for many other fauna including chipmunks, hummingbirds, and insects (Daily et al. 1993). Excavated cavities are important nesting habitats for a variety of vertebrate species including birds, mammals, amphibians, and reptiles (McComb and Noble 1981; Martin et al. 2004; Robles and Martin 2013), as well as myriad arthropods and fungi (Jackson and Jackson 2004; Tomás et al. 2008; Cockle et al. 2012).

Despite the functional role of red‐naped sapsuckers (hereafter, RNSA), relatively few studies have been conducted assessing their habitat relationships. Of the few studies, the majority have examined RNSA habitat selection within deciduous forests (Walters et al. 2002), which has indicated selection for mature aspen (e.g., *Populus tremuloides*) from the nest site to territory scale when aspen are available (Walters 1996; Walters et al. 2002; Sadoti and Vierling 2010). RNSA also occur, however, in conifer‐dominated forests where deciduous trees are largely absent (McClelland and McClelland 2000; Vierling et al. 2013), and less research has been conducted regarding habitat relationships of RNSA in these areas. Of the limited work conducted within conifer forests, RNSA appear to select large trees for nesting (McClelland and McClelland 2000) within the context of structurally diverse stands (Vierling et al. 2013). Additional research has been conducted within conifer forests on habitat relationships of other species related to RNSA; the red‐breasted sapsucker (*Sphyrapicus ruber*) and Williamson's sapsucker (*Sphyrapicus thyroideus*). Habitat relationships for these other species may be similar to RNSA in that large trees are preferred for nesting, but structural diversity is important for other activities such as foraging (Crockett and Hadow 1975; Bull et al. 1986; George et al. 2005; Wagner 2011). Additional research is clearly needed to understand the habitat relationships of RNSA within conifer‐dominated forests. This is particularly true given the steep historical and projected future declines in aspen (i.e., RNSA preferred habitat) across western North America (Frey et al. 2004; Worrall et al. 2008; Strand et al. 2009).

The ability to understand habitat relationships of single species, as well as species diversity, has been advanced in recent years with the incorporation of light detection and ranging (LiDAR) data (e.g., Nelson et al. 2005; Goetz et al. 2007; Vierling et al. 2008; Seavy et al. 2009; Vogeler et al. 2014; Zellweger et al. 2013; Davies and Asner 2014; Garabedian et al. 2014). LiDAR is used for mapping three‐dimensional vegetation structure at high resolution, which can be used in modeling frameworks to describe single‐scale or multiscale animal–environment relationships (e.g., Vierling et al. 2008; Davies and Asner 2014). While LiDAR data can be used to quantify traditional measures of vegetation that managers might use in habitat modeling (e.g., shrub density; Martinuzzi et al. 2009), LiDAR data can also be used to produce maps that show aspects of vegetation characteristics that are not easily measured in the field but nevertheless provide important perspectives on how vegetation structure influences animal populations and communities. For example, Weisberg et al. (2014) implemented a multiscale approach using LiDAR data to test MacArthur and MacArthur's (1961) hypothesis on the relationship between habitat structural heterogeneity and species diversity. An additional advantage of LiDAR includes the ability to vary the spatial scale of the study *post hoc* to explore the habitat variables that might be most influential to animals at different spatial scales (e.g., Seavy et al. 2009). Indeed, because LiDAR data provide 3D habitat information at both fine grain size and across large contiguous extents (Vierling et al. 2008), these approaches are powerful for building models that assess habitat relationships of animals.

Our objective was to characterize habitat relationships for RNSA in a conifer‐dominated forest using occupancy and LiDAR data, and to determine the extent(s) at which occupancy patterns were best explained. Occupancy during a bird's nesting season represents an area or territory that was accessible and selected (sensu Lele et al. 2013) based on reproductive requirements, which is thus a useful metric of inquiry when assessing habitat relationships. Based on previous work, our general hypothesis was that the most influential predictors of RNSA occupancy would include vegetation structure. We expected sapsuckers to select for structurally diverse areas to satisfy their requirements of foraging and nesting during the breeding season (Walters et al. 2002). Structurally diverse habitats might include 1) sparse large and mature trees for nest sites and drilling sapwells and 2) open canopy and shrubs for gleaning and flycatching activities as well as predator detection. Harvest activities within the study site generally leave seed trees, and thus, we also hypothesized that disturbance via harvest would positively influence occupancy of RNSA by creating heterogeneity in vegetation structure. We used three LiDAR metrics indicating forest structural characteristics, and one LiDAR‐derived and one non‐LiDAR‐derived metric to characterize disturbance. We selected three extents (2, 4, and 10 ha) to perform analyses, which were indicative of the range of territory sizes reported for RNSA (McClelland 1977; Walters et al. 2002).

This work builds on the analyses of Vierling et al. (2013), who implemented a methodological comparison of satellite and airborne LiDAR data to predict RNSA occupancy at a single and small extent (i.e., 0.32 ha). Our analyses are different in that we tested a priori hypotheses that describe RNSA habitat relationships across multiple extents. Our work addresses a large gap in knowledge regarding habitat selection of RNSA in conifer forests and has implications for management and conservation of an important ecosystem engineer.

## Methods

### Study area

**Figure 1:**
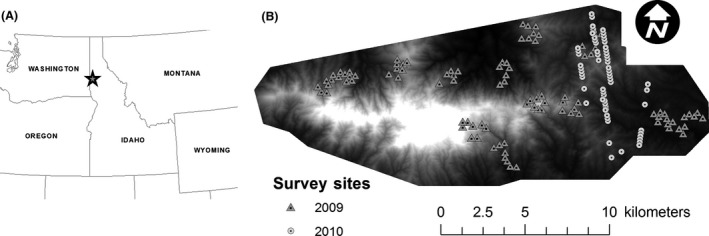
(A) Location of Moscow Mountain (i.e., star), Idaho, USA (Latitude: 46.803, Longitude: −116.868), where we surveyed for red‐naped sapsuckers (*Sphyrapicus nuchalis*) during 2009–2010. (B) Distribution of survey points by year across the study area with triangles and circles indicating 2009 and 2010, respectively. The map background is a digital terrain model derived from 2009 LiDAR data; light colors indicate high elevations (max = 1518 m); while darker colors indicate low elevations (min = 785 m).

Our study area was Moscow Mountain (Latitude: 46.803, Longitude: −116.868), northern Idaho, USA, which is privately and publicly owned and is actively managed (Fig. 1). Moscow Mountain is characterized as a mixed‐conifer forest, and the most abundant tree species include ponderosa pine (*Pinus ponderosa*), Douglas fir (*Pseudotsuga menziesii*), grand fir (*Abies grandis*), western red cedar (*Thuja plicata*), and western larch (*Larix occidentalis*). Elevation and precipitation range from 770 m to 1516 m and 630 to 1015 mm, respectively. Land ownership across the landscape is dominated by private timber companies interspersed with holdings of private and public land. Significant tree harvest occurred on Moscow Mountain between 2003 and 2009, which has been mapped (Hudak et al. 2012). Additional information about the study area can be found in Martinuzzi et al. (2009), Hudak et al. (2012), and Vierling et al. (2013).

### Woodpecker data

**Figure 2:**
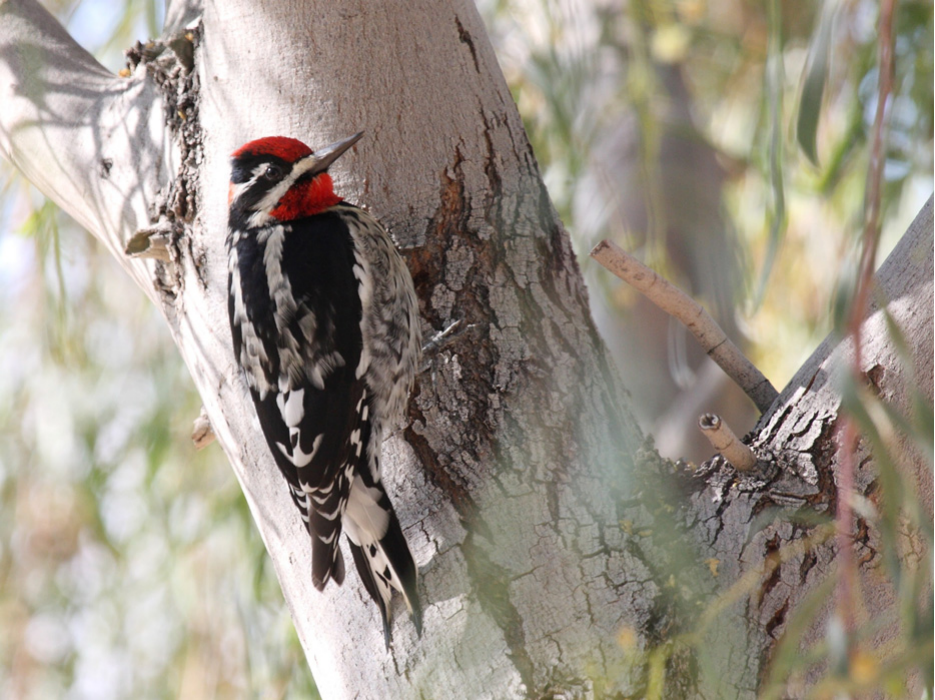
Red‐naped Sapsucker (*Sphyrapicus nuchalis*). We downloaded this photograph from Wikimedia Commons (Author: Dominic Sherony).

We surveyed for RNSA (Fig. 2) at 97 sites during 27 May–3 July in 2009 and at 65 sites during 19 May–2 July in 2010 (Fig. 1B). We implemented a stratified random design based on LiDAR‐derived canopy height (0–5 m, 6–15 m, and 16 m) and density metrics (0–40%, 41–70%, and 71–100%) for our 2009 sample to capture a range of vegetation structure (e.g., Vogeler et al. 2014). The spatially clustered nature of our sites in 2009 reflected broader geographical subunits that contained our target strata, but allowed us to maximize the number of sites sampled during the sampling season. Sites surveyed in 2010 were coincident with the footprint of the spaceborne Geoscience Laser Altimeter System (GLAS), which systematically sampled LiDAR data across the landscape along linear transects following its orbit around earth. We sampled within the GLAS footprint in 2010 because a broader goal of our research was to assess the differences between satellite and airborne derived LiDAR data when applied to animal habitat modeling (Vierling et al. 2013). To guard against double‐counting the same birds, we selected sites with a minimum of 340 m spacing (~10 ha area between survey locations), which corresponds to the larger territory sizes reported for RNSA (McClelland 1977; Walters et al. 2002). We constrained our dates to correspond to the breeding season of RNSA. To elicit RNSA responses, we implemented the protocol of Drever et al. (2008) using playback recordings. We played two identical playbacks (i.e., call and drum) with a 2 min silence in between. At each site, we recorded the occupancy (i.e., 0 or 1) of RNSA. It is important to note that playback recordings are only effective during the breeding season when individuals are territorial (Walters et al. 2002), which is why we temporally constrained our sampling. Further, we surveyed each site twice within the breeding season to account for imperfect detection. Detection bias is an issue when the probability of detection is less than one, and in such situations, estimates of occupancy can be biased (Gu and Swihart 2004; Guillera‐Arroita et al. 2014).

### Explanatory variables

We compiled five environmental metrics describing vegetation structure and disturbance at three different spatial extents including the density of the upper canopy (hereafter upper canopy density), shrub density, canopy height diversity, distance to roads, and area harvested (Table 1). Four of the five descriptors were derived from LiDAR data, while one (i.e., distance to roads) was derived from county road information (Table 1). We summarized metrics (except distance to roads) at 2‐, 4‐, and 10‐ha buffers around survey points (Table 2). We selected these extents because they cover the range of territory sizes reported for RNSA during the breeding season (McClelland 1977; Walters et al. 2002).

**Table 1:** Description of metrics used as explanatory variables (with codes) at 2‐, 4‐, and 10‐ha extents for modeling occupancy of red‐naped sapsuckers (*Sphyrapicus nuchalis*) during 2009–2010 at Moscow Mountain, Idaho, USA. Metrics were calculated at 20 m resolutions

Category	Variable (code – units)	Description
Vegetation structure	Upper canopy density (tree – summed %)	LiDAR metric indicating sum (across 20 × 20 m pixels) of percentage of laser returns within >20 m and ≤30 m in height. We used this as an index of large tree density or forest maturity. Data were collected in 2009.
	Canopy height diversity (HSD – m)	LiDAR metric indicating SD (across 20 × 20 m pixels) in mean height of canopy. We used this as an index of foliage height diversity (MacArthur and MacArthur 1961). Data were collected in 2009.
	Shrub density (shrub – summed %)	LiDAR metric indicating sum (across 20 × 20 m pixels) of percentage of laser returns within > 1 m and ≤ 2.5 m in height. We used this as an index of shrub density. Data were collected in 2009.
Disturbance	Area harvested (harvest – m^2^)	Area classified as losing greater than 66 Mg/ha of biomass from 2003 to 2009 (Hudak et al. 2012). Predictions were based on 2003 and 2009 LiDAR data.
	Distance to roads (road – m)	Mean distance to county roads in Latah County, Idaho. County roads were used at the 1:250,000 scale. Raster data indicating distance to road were at 20 m resolutions.

**Table 2:** Mean (and range) of explanatory variables at 2‐, 4‐, and 10‐ha extents used in modeling occupancy of red‐naped sapsuckers (*Sphyrapicus nuchalis*) during 2009–2010 at Moscow Mountain, Idaho, USA. Variable descriptions are as follows: shrub density – sum of percentage of vegetation returns >1 m and ≤2.5 m in height, canopy height diversity – standard deviation of canopy heights, and upper canopy density – sum of percentage of vegetation returns > 20 m and ≤30 m in height, area harvested – area (m^2^) harvested. Values are not standardized

Extent	Shrub density	Canopy height diversity	Upper canopy density	Area harvested
2 ha	329 (32–1335)	3.16 (0.42–7.36)	598 (0–2564)	4783 (0–20,800)
4 ha	617 (85–2010)	3.46 (0.79–9.09)	1146 (0–4656)	9580 (0–40,000)
10 ha	1383 (108–3933)	3.67 (0.46–8.95)	2476 (0–11,250)	24,140 (0–101,600)

LiDAR data were collected for Moscow Mountain during the summer of 2009. Details on data acquisition and processing are described in Hudak et al. (2012) and Vierling et al. (2013). The vegetation variables used in this study were computed for LiDAR data sets using the height, density, and intensity of LiDAR returns within 20 m square grid cells across the study area. We selected our subset of variables due to their potential ecological importance (Table 1). First, we expected RNSA to be negatively associated with upper canopy density; RNSA are noted to employ flycatching as a foraging method (Walters et al. 2002), and a dense upper canopy would likely inhibit this activity. Additionally, we hypothesized that RNSA would select territories that exhibited high diversity in vegetation structure (i.e., a combination of tall and short vegetation elements). RNSA require large trees for nest sites, and some sapwells have been noted to be in large trees (trees >25 cm dbh; Walters et al. 2002). However, shorter vegetation canopies would also be important because they might represent open areas that could be used as flycatching habitat (Walters et al. 2002). Both nesting activities and foraging activities would therefore occur within territories exhibiting high canopy diversity, which is why we predicted a positive effect of canopy height diversity. Finally, although a dense shrub layer might provide a foraging base for flying insects, we hypothesized that RNSA would be negatively associated with shrub density because a more open understory would seemingly provide more opportunities for birds to detect insects during foraging, as well as increase detection of predators (Li and Martin 1991). This hypothesis was also consistent with RNSA selecting areas with high canopy height diversity, rather than areas with monocultures of shrubs. We used zonal statistics in ArcGIS 10.1 (ESRI 2012) to generate measurements of aforementioned variables from the rasterized 20 m LiDAR metrics, with 2, 4, or, 10 ha polygons surrounding sample points constituting the zones.

Additionally, we used a LiDAR‐derived raster indicating area harvested (Table 1), which was developed by Hudak et al. (2012). Hudak et al. (2012) characterized presence/absence of harvest activity by comparing the estimated change in aboveground tree biomass mapped from independent field and LiDAR surveys in 2003 and 2009. Tree biomass in both 2003 and 2009 was predicted from LiDAR metrics and machine learning (i.e., Random Forest; Breiman 2001) algorithms (Hudak et al. 2012). Hudak et al. (2012) reported a biomass change of < −66 Mg/ha between 2003 and 2009 as indicative of harvest activity based on field validation and stand maps of harvest units provided by forest industry partners. We used the raster for area harvested to determine the influence of harvest on RNSA occupancy. We expected RNSA to be positively associated with area harvested because it generates structural diversity and edges, which could provide foraging space for flycatching or gleaning (Walters et al. 2002). We used the tabulate area tool in ArcGIS 10.1 (ESRI 2012) to generate values of area harvested.

Finally, we used a county road layer for Latah County, Idaho, which included Moscow Mountain, to develop a “distance to roads” raster (mean = 353, range = 28–1348). We used the distance tool in ArcGIS to develop the raster at a 20 m resolution. We expected RNSA occupancy to be negatively associated with distance to roads because roads may provide edges and open space for foraging activities. However, Walters et al. (2002) indicated that RNSA are affected little by traffic, thus we might not observed a detectable effect of roads. It is important to note that a positive association with distance to road indicates occupancy away from roads, whereas a negative association indicates occupancy near roads. We used zonal statistics in ArcGIS 10.1 (ESRI 2012) to calculate the mean distance to road.

### Statistical modeling

Prior to model development, we assessed multicollinearity between our explanatory variables. Results indicated that our variables were uncorrelated (¦r¦ < 0.64), and thus we retained all of them for building our RNSA occupancy models. To build our occupancy models, we implemented the approach of MacKenzie et al. (2002, 2006) using the R package “unmarked” (Fiske and Chandler 2011; R Core Team 2013). This approach allowed us to maximize the likelihood of occupancy (*ψ*), while accounting for imperfect detection (*p*), given detection histories over the sites surveyed (MacKenzie et al. 2006).

We built null, subset, and global models, and our subset and global models reflected our hypotheses about the influence of vegetation structure and disturbance on occupancy of RNSA. Because we surveyed different sites in 2009 and 2010, we included an effect of survey year in all models to account for an additive difference in occupancy among years. Modeled parameters were estimated using maximum likelihood, and we evaluated the influence of our standardized covariates on *ψ* using the logit link function. We standardized our covariates to assist with likelihood convergence. Because previous modeling efforts with RNSA detection data indicated detection probability did not significantly vary with survey‐specific covariates (see Vierling et al. 2013), we did not include any effects on detection. We did, however, assess an effect of year on detection probability, but in all instances, it was less supported relative to models without a year effect (based on Akaike's information criterion); thus, we excluded a year effect on detection. We modeled detection probability assuming it was less than one, and constant among sites, surveys, and years.

We evaluated our competing models using Akaike's information criterion adjusted for small sample size (AIC_c_; Burnham and Anderson 2002) using the R package “AICcmodavg” (Mazerolle 2014). The model with the smallest AIC_c_ is most supported; however, when ΔAIC_c_ < 2 multiple models are supported by the data (Burnham and Anderson 2002). Because model support changed with extent of analysis (see Results), we elected to display both top model and model‐averaged parameter estimates to characterize the gradient in parameter effect size and precision. Model‐averaged parameter estimates are simply a sum of the product of parameter estimates and their associated model weight across all models. Following the suggestions of MacKenzie and Bailey (2004), we evaluated fit of our global model using a parametric bootstrap approach, where we compared our observed chi‐squared (*X* ^2^_O_) statistic to our bootstrapped (*X* ^2^_B_) value to determine the probability of observing a larger value. We implemented this test using the “AICcmodavg” package in R (Marzerolle 2014; R Core Team 2013) and used 1000 bootstrapped samples. We considered *P*‐values > 0.05 to indicate adequate model fit.

Finally, modeling species‐environment relationships can suffer from issues of spatial autocorrelation (Lichstein et al. 2002). Thus, we examined residual spatial autocorrelation among our top models at each extent. We estimated Moran's *I* values using inverse distance weighting within the “ape” package in R (Paradis et al. 2004; R Core Team 2013). Moran's *I* ranges between −1 and 1 indicating perfect dispersion and perfect correlation, respectively. We assumed that our top models removed spatial autocorrelation if they appropriately captured factors influencing RNSA occupancy. However, if residual spatial autocorrelation was high, we deduced that our models excluded factors that were likely important for describing RNSA occupancy.

## Results

The distance to a RNSA detection was relatively short and consistent, while considerable variation in occupancy was observed. The maximum distance to a detected RNSA was ~300 m with a mean distance of ~39 m. We detected RNSA one of the two surveys at 49 locations (30%), twice at 18 locations (11%), or not at all at 95 locations (59%), which resulted in a naïve occupancy estimate of 0.41 across our survey locations.

Model selection uncertainty was highest at the 2‐ha extent, whereas results were generally consistent at the 4‐ and 10‐ha extents. At the 2‐ha extent, four models exhibited a ΔAIC_c_ < 2 and displayed a range in parameters (i.e., 4–8; Table 3). The top model included an effect of year and area harvested on occupancy, but the global model was also within the suite of supported models (Table 3). At the 4‐ha extent, only two models exhibited a ΔAIC_c_ < 2 and the top model was the global model, which contained the year effect as well as all effects of vegetation structure and disturbance on occupancy (Table 3). The second ranked model was the same as the global model, but lacked the effect of distance to roads. At the 10‐ha extent, model selection results were similar to the patterns observed at the 4‐ha extent; however, the top model did not contain the effect of distance to roads on occupancy (Table 3). The second supported model contained the effect of distance to roads; however, in this case, it was a “pretending variable” because it did not add any substantive information relative to the model without the road effect (Anderson 2008). Considering all models across all extents, the most supported model was the global model at the 4‐ha extent because it exhibited the lowest overall AIC_c_ value (350.48; Table 3).

**Table 3:** Hypotheses and model selection results for models assessing occupancy of red‐naped sapsuckers (*Sphyrapicus nuchalis*) models during 2009–2010 at 2‐, 4‐, and 10‐ha extents at Moscow Mountain, Idaho, USA. The number of estimated parameters and Akaike weights for each model are indicated by *K* and w_i_, respectively

Hypothesis	Model	*K*	AIC_c_	ΔAIC_c_	*w*_i_
	2 ha				
Harvest	*ψ*(year + harvest), *p*(.)	4	358.75	0.00	0.35
Disturbance	*ψ*(year + harvest + road), *p*(.)	5	359.27	0.51	0.27
Vegetation structure + disturbance	*ψ*(year + shrub + tree + HSD + harvest + road), *p*(.)	8	359.85	1.10	0.20
Vegetation structure + harvest	*ψ*(year + shrub + tree + HSD + harvest), *p*(.)	7	360.57	1.82	0.14
Null	*ψ*(year), *p*(.)	3	364.18	5.42	0.02
Vegetation structure	*ψ*(year + shrub + tree + HSD), *p*(.)	6	364.30	5.54	0.02
	4 ha				
Vegetation structure + disturbance	*ψ*(year + shrub + tree + HSD + harvest + road), *p*(.)	8	350.48	0.00	0.51
Vegetation structure + harvest	*ψ*(year + shrub + tree + HSD + harvest), *p*(.)	7	351.16	0.68	0.36
Harvest	*ψ*(year + harvest), *p*(.)	4	354.43	3.95	0.07
Disturbance	*ψ*(year + harvest + road), *p*(.)	5	355.28	4.80	0.05
Vegetation structure	*ψ*(year + shrub + tree + HSD), *p*(.)	6	359.22	8.74	0.01
Null	*ψ*(year), *p*(.)	3	364.18	13.70	0.00
	10 ha				
Vegetation structure + harvest	*ψ*(year + shrub + tree + HSD + harvest), *p*(.)	7	355.89	0.00	0.51
Vegetation structure + disturbance	*ψ*(year + shrub + tree + HSD + harvest + road), *p*(.)	8	357.44	0.33	0.44
Harvest	*ψ*(year + harvest), p(.)	4	356.42	5.55	0.03
Disturbance	*ψ*(year + harvest + road), *p*(.)	5	358.18	6.69	0.02
Vegetation structure	*ψ*(year + shrub + tree + HSD), *p*(.)	6	363.98	9.82	0.00
Null	*ψ*(year), *p*(.)	3	365.15	12.81	0.00

**Figure 3:**
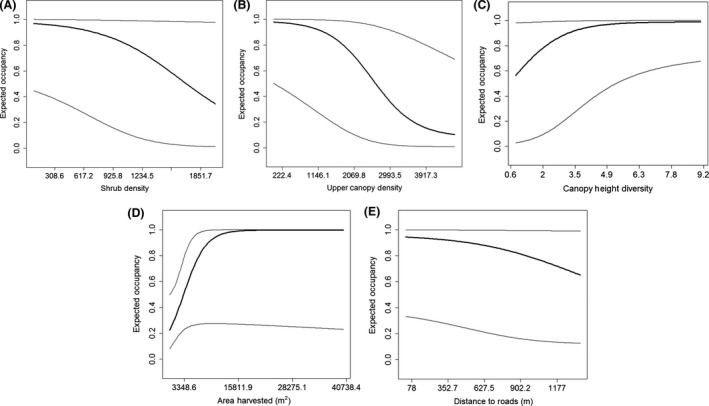
Model‐averaged relationships between predicted occupancy (*ψ*) and vegetation structure (A – shrub density, B – canopy density, C – canopy height diversity) and disturbance (D – area harvested, E – distance to roads) for red‐naped sapsuckers (*Sphyrapicus nuchalis*) at the 4‐ha extent at Moscow Mountain, Idaho, USA. Dark lines indicate predicted values, and gray lines are 95% CIs. Units on the *x*‐axis are original values (i.e., not standardized).

The direction of effect for all variables was similar across spatial extents, but the effect size increased with spatial extent and statistical significance peaked at the 4‐ha extent (Tables 4 and 5). The effect of year on occupancy was negative indicating that RNSA occupancy at the sites visited in 2010 was lower than occupancy in 2009; however, the effect only approached statistical significance at the 4‐ha extent (Table 4). Shrub density and upper canopy density both had a negative effect on RNSA occupancy (Fig. 2), and the effect was generally supported across top model and model‐averaged estimates at the 4‐ha extent (Tables 4 and 5). Canopy height diversity and area harvested both had a positive effect on RNSA occupancy (Fig. 2 and Table 4), but canopy height diversity was the only model‐averaged effect that was supported at the 4‐ha extent (Table 5). Finally, the effect of roads on RNSA occupancy was negative indicating a positive relationship between occupancy and roads (Fig. 3), but the effect was only weakly supported (Tables 4 and 5).

**Table 4:** Top model parameter estimates (standardized) describing occupancy of red‐naped sapsuckers (*Sphyrapicus nuchalis*) during 2009–2010 at 2‐, 4‐, and 10‐ha extents at Moscow Mountain, Idaho, USA. Variable descriptions are as follows: year – indicator variable (0 = 2009, 1 = 2010), shrub density – sum of percentage of vegetation returns >1 m and ≤2.5 m in height, canopy height diversity – standard deviation of canopy heights, upper canopy density – sum of percentage of vegetation returns >20 m and ≤30 m in height, area harvested – area (m^2^) harvested, and distance to roads – mean (m) distance to roads at the 2‐ha extent

	2 ha			4 ha			10 ha		
	Standardized coefficient	SE	*P*‐value	Standardized coefficient	SE	*P*‐value	Standardized coefficient	SE	*P*‐value
Intercept	1.11	1.00	0.27	4.65	2.16	0.03^b^	4.97	4.09	0.22
Year	−0.73	0.57	0.20	−2.09	1.10	0.06^a^	−3.17	2.13	0.14
Shrub density	–	–	–	−0.81	0.45	0.07^a^	−1.24	1.03	0.23
Upper canopy density	–	–	–	−1.77	0.71	0.01^b^	−2.78	1.80	0.12
Canopy height diversity	–	–	–	1.27	0.65	0.05^b^	2.92	2.23	0.19
Area harvested	1.14	1.20	0.31	5.25	2.74	0.06^a^	3.79	3.31	0.25
Distance to roads	–	–	–	−0.74	0.48	0.12	–	–	–

a *P*‐value ≤ 0.10.

b *P*‐value ≤ 0.05.

**Table 5:** Model‐averaged parameter estimates (standardized) describing occupancy of red‐naped sapsuckers (*Sphyrapicus nuchalis*) during 2009–2010 at 2‐, 4‐, and 10‐ha extents at Moscow Mountain, Idaho, USA. Variable descriptions are as follows: year – indicator variable (0 = 2009, 1 = 2010), shrub density – sum of percentage of vegetation returns >1 m and ≤2.5 m in height, canopy height diversity – standard deviation of canopy heights, upper canopy density – sum of percentage of vegetation returns >20 m and ≤30 m in height, area harvested – area (m^2^) harvested, and distance to roads – mean (m) distance to roads at the 2‐ha extent

	2 ha			4 ha			10 ha		
	Standardized coefficient	SE	95% CI	Standardized coefficient	SE	95% CI	Standardized coefficient	SE	95% CI
Intercept	1.02	0.83	−0.61, 2.66	4.17	2.20	−0.14, 8.47	4.01	3.26	−2.37, 10.39
Year	−0.82	0.59	−1.98, 0.34	−1.72	1.07	−3.81, 0.38	−2.89	1.82	−6.46, 0.68
Shrub density	−0.24	0.29	−0.81, 0.32	−0.75	0.43	−1.60, 0.09	−1.06	0.86	−2.75, 0.63
Upper canopy density	−0.65	0.34	−1.33, 0.02	−1.66	0.68	−2.99, −0.33^a^	−2.47	1.49	−5.39, 0.46
Canopy height diversity	0.56	0.35	−0.13, 1.25	1.29	0.67	−0.02, 2.59	2.41	1.86	−1.22, 6.05
Area harvested	1.03	0.89	−0.72, 2.77	4.84	2.76	−0.56, 10.25	3.14	2.62	−1.99, 8.28
Distance to roads	−0.44	0.32	−1.07, 0.18	−0.72	0.48	−1.67, 0.23	−0.69	0.45	−1.58, 0.19

a 95% CI does not overlap 0.

Assessments of model fit, detection probability, and residual autocorrelation indicated consistent results across spatial extents. Goodness‐of‐fit statistics suggested appropriate fit for all global models (*X* ^2^ = 0.50–0.89, *P *=* *0.75–0.91), and in all cases, more complex models were supported relative to the null model indicating our data were informative. Detection probabilities from top models were similar (*P* = 0.36–0.42, SE = 0.04–0.07), and we observed no evidence of residual spatial autocorrelation in our top models (Moran's *I *=* *−0.1–0.00, *P *=* *0.67–0.96).

## Discussion

Ecosystem engineers are functionally important within ecological communities (Daily et al. 1993; Jones et al. 1994; Robles and Martin 2013), and thus, understanding factors that influence their occupancy is essential to understanding the ecological integrity of a site. Using LiDAR data, we characterized how vegetation structure and disturbance influence the occupancy of the red‐naped sapsucker, an ecosystem engineer, in northern Idaho. Our results indicated that the 4‐ha extent was the most informative for characterizing RNSA occupancy within our coniferous study area. Further, we discovered that the density of shrubs and large trees negatively affects RNSA occupancy, while canopy height diversity positively affected occupancy. These findings suggest moderate territory sizes (~4 ha) for red‐naped sapsuckers in our study area, and that vegetation structure largely influences where territories are placed on the landscape.

Our results were generally consistent with our expectations concerning the effect of vegetation structure on occupancy of RNSA. First, we predicted that RNSA would be negatively associated with shrub density because of impairments to foraging via flycatching and gleaning, as well as potential reduction in predator detection. Our results supported this prediction in that we observed a negative relationship between occupancy and shrub density. Second, we predicted that sapsuckers would be negatively associated with upper canopy density because of fewer open areas for foraging, and our results indicated a negative association. Third, we suggested that sapsucker occupancy would be positively associated with our index of foliage height diversity (MacArthur and MacArthur 1961) because canopy height diversity would provide a wide distribution of resources for RNSA (nest sites, sapwells, foraging space; Walters et al. 2002). Indeed, our results were consistent the general hypothesis that RNSA territories are associated with areas that are structurally diverse, and exhibit a sparse distribution of large trees and an open shrub layer. This type of forest structure provides nesting and foraging habitat for RNSA (McClelland and McClelland 2000; Walters et al. 2002), as well as closely related sapsucker species (Crockett and Hadow 1975; Bull et al. 1986; George et al. 2005; Wagner 2011).

Similarly, we expected sapsuckers to be positively associated with area harvested and negatively associated with distance to roads because harvest and roads both generate landscape diversity. We hypothesized RNSA would use these open areas for foraging activities. Model selection results supported these predictions in that area harvested was in the top model at all extents, and distance to roads was in the top model at the 4‐ha extent (Table 3). These results support previous studies indicating RNSA can be abundant and successfully reproduce in logged areas (Tobalske 1992). Furthermore, Ortega and Capen (2002) noted that there was no difference in yellow‐bellied sapsucker (*Sphyrapicus varius*) abundance between interior sites and sites near roads. Despite the model selection results, however, model‐averaged parameter estimates indicated weak associations between area harvested, distance to roads, and occupancy (Table 5). Overall, our analyses indicated that vegetation structural characteristics and disturbance both influenced RNSA occupancy, but vegetation structure was the more influential factor.

A growing list of studies use LiDAR to describe animal–habitat relationships (see reviews by Hill et al. 2014; Davies and Asner 2014; Mueller and Vierling 2014), and the use of LiDAR allowed us to explore the relative effects of vegetation structure on RNSA occupancy in coniferous forests across 3 spatial extents. The flexibility to explore spatial extents *post hoc* is powerful (e.g., Seavy et al. 2009), and through this flexibility, we identified the extent of forest structural characteristics that provided the best fit for our RNSA occupancy data. Indeed, LiDAR data in our context provided a unique opportunity to quantify and assess the influence of forest canopy characteristics (that are otherwise difficult to measure) at relatively broad spatial extents. Other studies have noted that forest canopy characteristics derived from LiDAR improved their understanding concerning patterns of wildlife occupancy. For instance, Vogeler et al. (2014) found that upper canopy density influenced occupancy of brown creepers (*Certhia americana*), and Palminteri et al. (2012) found that upper canopy characteristics influenced occupancy of bald‐faced saki monkeys (*Pithecia irrorata*) in Amazonian rainforests. Measures of canopy structural variability have also been noted to influence occupancy of black‐throated blue warblers (*Dendroica caerulescens;* Goetz et al. 2010). The ability to map forest structural characteristics from the upper canopy to the low understory using LiDAR has, and will continue to, advanced our ability to better understand habitat relationships of animals, as well as extrinsic drivers of population fitness (e.g., survival and reproduction).

### Conservation implications

This study provides an example of how LiDAR data can be used to understand animal–environment relationships in a multiscale framework. Our analyses identified the relative influence of vegetation structure and disturbance on RNSA occupancy in a conifer‐dominated forest. RNSA fill an important ecological role in that they create resources important for other species (Daily et al. 1993; Robles and Martin 2013). Because of their role, it may be of interest for forest managers to incorporate RNSA ecology when making decisions. If RNSA are considered to be important, we suggest managers focus on structural diversity (e.g., moderate–low shrub density, variability in canopy layers; Fig. 3) at intermediate territory sizes (e.g., 4–6 ha). Based on our data, this would be a conservative approach to manage for the conservation of RNSA in mixed‐conifer forests similar to those on Moscow Mountain, Idaho, USA. It would, however, be critical to conduct additional work to understand the influence of landscape metrics on RNSA fitness (i.e., reproductive success, survival, or population growth).

Finally, as aspen stands continue to decline throughout western North America (Frey et al. 2004), information on aspen‐associated animals within systems lacking aspen will be increasingly important for conservation and management. For example, by assessing habitat relationships of animals abundant within and outside of aspen‐dominated areas, such as RNSA, managers could better evaluate management options given aspen declines that might occur in the foreseeable future (Rehfeldt et al. 2009; Strand et al. 2009). This premise does not suggest that conifer‐dominated areas replace aspen stands, rather it advocates for information on habitat relationships in both aspen and nonaspen locations. This information would provide managers the opportunity to make proactive decisions in the midst of current and broadscale ecological changes, leading to proactive rather than reactive wildlife conservation.

## Conflict of Interest

None declared.
